# U-shaped relationship between frailty and non-HDL-cholesterol in the elderly: a cross-sectional study

**DOI:** 10.3389/fnut.2025.1596432

**Published:** 2025-05-21

**Authors:** Yu Pan, Yan Yuan, Juan Yang, Zhu Qing Feng, Xue Yin Tang, Yi Jiang, Gui Ming Hu, Jiang Chuan Dong

**Affiliations:** ^1^Department of Geriatrics, The Second Affiliated Hospital of Chongqing Medical University, Chongqing, China; ^2^Department of Integrated of Chinese and Western Medicine, The First Affiliated Hospital of Chongqing Medical University, Chongqing, China; ^3^Oncology Treatment Center of Traditional Chinese Medicine, Affiliated Cancer Hospital of Chongqing University, Chongqing, China

**Keywords:** frailty, non-HDL-cholesterol, cross-sectional study, CHARLS, NHANES

## Abstract

**Background:**

The modulation of lipid metabolism has been explored as a potential treatment for frailty, yet the association between non-high-density lipoprotein-cholesterol (non-HDL-C) and frailty remains unclear.

**Methods:**

This study utilized data from five cycles of the National Health and Nutrition Examination Survey (NHANES) and two cycles of the China Health and Retirement Longitudinal Study (CHARLS) to investigate this relationship. A 40-item frailty index scale, encompassing various dimensions of somatic functioning, psychological evaluation, and illness, was developed and individually evaluated for each participant. The variables underwent screening through Least Absolute Shrinkage and Selection Operator (LASSO) regression, univariate logistic regression, and Light Gradient Boosting Machine (LightGBM), with models developed through multivariate logistic regression and the LightGBM algorithm. Subsequently, subgroup analyses and interaction tests were conducted to substantiate correlations.

**Results:**

The U-shaped nonlinear association between non-HDL-C and frailty in older adults was validated using the LightGBM algorithm. Non-HDL cholesterol levels in the range of 117.54–194.64 mg/dL were less likely to be frailty, while the likelihood of developing frailty was higher at 47.99–63.87 or 274.01–259.65 mg/dL. Subgroup analyses and interaction tests confirm these results.

**Conclusion:**

It is plausible that an intricate nonlinear association between non-HDL-C and frailty in the elderly exists, though further rigorously designed studies are imperative to validate this relationship.

## 1 Introduction

Frailty is a multifaceted age-related clinical condition marked by diminished physiological reserves and heightened susceptibility to internal and external stressors, involving dysfunction in the neuromuscular, metabolic, and immune systems (1–3). Various assessment instruments are utilized in the identification of frailty, with the Frailty Phenotype and Deficiency Accumulation Approach being acknowledged as two credible methodologies. The concept of “Frailty Phenotype” was introduced by Fried and his colleagues, encompassing five physical criteria: unintentional weight loss, self-reported fatigue, weakness, slow gait speed, and low physical activity, with the presence of three or more indicating frailty (4). The cumulative deficits method is utilized to calculate a frailty index (FI), which is a continuous score representing the number of health deficits present in an individual relative to the total number of items evaluated, with values ranging from 0 (indicating no deficits) to 1 (indicating the presence of all deficits), thus reflecting the individual’s level of frailty (5, 6). Interestingly, despite variations in the specific deficits included and the number of deficits considered in studies proposing a FI, the results obtained from the index consistently demonstrate statistical consistency (7, 8).

Accumulating research has increasingly identified a range of reliable biomarkers associated with frailty, including oxidative stress imbalance and inflammatory markers (9). Teixeira-Gomes et al. demonstrated that levels of inflammatory mediators (CRP and IL-6) and oxidized DNA were significantly elevated in adults classified as frail according to the Fried criteria, compared to their non-frail counterparts (10). Additionally, another research reported that in patients with cerebrovascular disease and cognitive frailty, lipid peroxidation, indicated by increased MDA levels, was elevated, while superoxide dismutase (SOD) activity was reduced, alongside heightened levels of inflammatory markers such as CRP, IL-6, and TNF-*α* (11). Moreover, Hammami et al. identified a positive correlation between the frailty index and inflammatory markers CRP, IL-6, IL-8, and TNF-α (12). Notably, after adjusting for age, the association between CRP and frailty remained robust and significant. Furthermore, a meta-analysis conducted by Mailliez et al. revealed that, in addition to CRP, four other biomarkers—vitamin D, albumin, hemoglobin, and free testosterone—were also significantly associated with frailty (13).

Additionally, research has indicated a correlation between frailty and long-term dietary habits, indicating a potential connection between frailty and metabolomics (14). The study of lipid metabolism in older frail or pre-frail individuals has been a significant focus of research (15–17). Lipids, particularly low-density lipoprotein cholesterol (LDL-C), have been shown to contribute to atherosclerosis, oxidative stress, and accelerated senescence of endothelial progenitor cells through various modified forms such as oxidized LDL, acetylated LDL, ethylated LDL, methylated LDL, and glycosylated LDL (18). HDL, on the other hand, is considered the ‘body protector’ of lipids because of its antioxidant, antithrombotic, anti-inflammatory and anti-apoptotic properties (19). Interestingly, previous studies have yielded conflicting findings regarding the role of lipid compounds as biomarkers in frailty (20–23). For instance, a study conducted by Tavares and his colleagues found no correlation between high-density lipoprotein (HDL) levels and frailty in the elderly (24), while Lina Ma and her team determined that frail older adults had lower levels of HDL and hemoglobin compared to their non-frail counterparts (21). Such inconsistent results emphasize the necessity for additional research. A study evaluating the oxidant-antioxidant balance in elderly individuals by measuring varying concentrations of HDL-C revealed that participants in the high HDL-C group exhibited lower triglyceride concentrations, whereas those in the low HDL-C group demonstrated elevated levels of oxidative stress (22). It is posited that an imbalance in oxidative stress may constitute a critical mechanism through which lipid metabolism affects the frailty status of individuals.

Recently, the concept of non-HDL-C has been introduced by researchers as a comprehensive measure of cholesterol across various lipoproteins excluding HDL. Non-HDL-C is calculated based on lipid distribution, with its specific value derived by subtracting the HDL-C level from the total cholesterol level measured in the body. A growing body of evidence corroborates the association of non-HDL-C with atherosclerotic dyslipidemia linked to metabolic disorders, type 2 diabetes mellitus, and obesity, further establishing it as a more robust predictor of risk for lipid-related diseases (25–27). The atherogenic properties of non-HDL-C indicate its potential utility as a biomarker for predicting adverse cardiovascular events that could potentially exacerbate the onset of frailty in the elderly through its impact on cardiovascular health and other physiological pathways (28). Nevertheless, there is currently a lack of research investigating the association between non-HDL-C levels and frailty.

To address this research gap, we developed a 40-item frailty index (FI) scale following the established development steps (29). Subsequently, we utilized datasets from various public databases and applied machine learning algorithms to investigate the following specific research inquiries (30). Initially, traditional statistical methods were utilized to investigate the impact of the FI in conjunction with non-HDL-C. Subsequently, an analysis was conducted utilizing the sample data to examine the correlation between frailty and non-HDL-C. The variables were then prioritized based on their significance using the Light Gradient Boosting Machine (LightGBM) algorithm, a variant of the GB algorithm created by Microsoft, renowned for its exceptional efficacy in handling extensive structured datasets and rapid training capabilities (31, 32). Moreover, polynomial modeling was conducted with LightGBM to further elucidate the intricate association between non-HDL-C and frailty in the elderly. The overarching objective is to offer personalized intervention strategies based on the levels of non-HDL-C in older frail patients.

## 2 Methods

### 2.1 Study design

A research program called the National Health and Nutrition Examination Survey (NHANES) is structured to evaluate the health and nutritional well-being of both adults and children residing in the United States. This program employs a combination of questionnaires and physical examinations to target specific demographic groups or health-related issues. Another study, the China Health and Retirement Longitudinal Study (CHARLS), serves as a longitudinal survey representative of individuals aged 45 years and older in mainland China (33). The objective is to construct a high-quality public micro-database encompassing multidimensional data on socioeconomic and health status, to fulfill the requirements of scientific research on aging.

**Figure 1:**
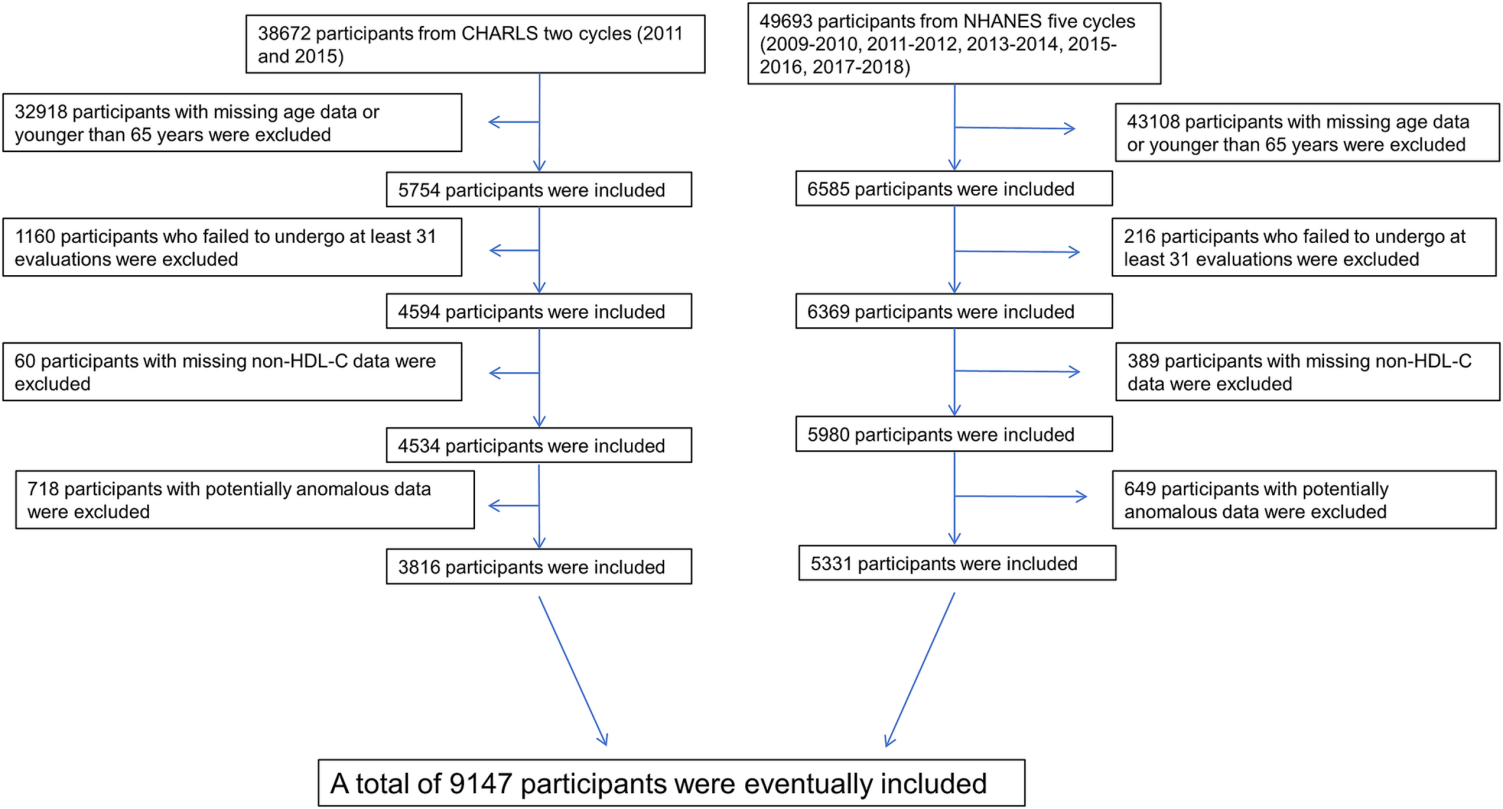
The participants’ selection process.

This study initially enrolled 88,365 participants from multiple survey cycles, including the NHANES surveys from 2009 to 2010, 2011 to 2012, 2013 to 2014, 2015 to 2016, 2017 to 2018, and the CHARLS 2011 and 2015 surveys. According to the standards of the World Health Organization and the United Nations, people over 65 years old are defined as the elderly. Participants with missing or potentially anomalous relevant data or below the age of 65 were excluded. Additionally, it has been shown in prior research that despite potential missing data among participants, frailty can still be identified through a minimum of 75% FI assessments, even if the specific correlates of frailty may vary. As a result, individuals with a significant amount of missing data and who did not complete at least 75% FI assessments were excluded from the study (7, 8). Ultimately, a total of 9,147 participants were included in the study (Figure 1).

The variables considered for each case encompassed patient demographics such as gender, age, race, and education, indicators from pertinent laboratory tests including total cholesterol, HDL data, blood urea nitrogen (BUN), serum glucose, participants’ body mass index (BMI) calculated as weight divided by the square of height (kg/m^2^) (34), and a total of 20 variables were ultimately included.

### 2.2 Frailty assessment

Based on the foundational criteria and procedures for constructing the FI, as well as incorporating data from the NHANES and CHARLS, a 40-item assessment scale was devised for evaluating the frailty condition of individuals in this research (22). This scale aims to comprehensively evaluate multiple dimensions of health, illness, physical functioning, and mental well-being to assess the extent of frailty in each participant. Utilizing the FI scale and drawing upon existing literature, we further categorized three levels of frailty: absence of frailty (FI ≤ 0.12), pre-frailty (0.12 < FI < 0.25), and frailty (FI ≥ 0.25) (29, 35, 36). A comprehensive account of the item composition and scoring criteria for the FI scale is available in Supplementary Table 1.

### 2.3 Non-HDL-C

Non-HDL-C comprising all plasma lipoproteins exclusive of HDL-C, is determined by subtracting HDL-C from total cholesterol. Blood test data from the CHARLS database were centrally examined by the Youanmen Clinical Laboratory Center of Capital Medical University using the enzymatic colormetric test. Two staff members from the Chinese Center for Disease Control and Prevention were responsible for the storage of blood samples on a full-time basis. Quality control samples were used daily in the laboratory during the testing process. The coefficient of variation was not more than 1.0% for within-assay and 1.7% for between-assay. Serum samples from the NHANES database are frozen and stored after collection and then sent to CDC/NCEH/DLS for examination. As part of the routine serum biochemical analyses, various lipid levels were measured using the Beckman Coulter UniCel® DxC800.The coefficients of variation for both the within- and between-assay were controlled as well.

Non-HDL-C comprising all plasma lipoproteins exclusive of HDL-C, is determined by subtracting HDL-C from total cholesterol. Because of the many factors influencing non-HDL-C, in this study we excluded data such as smoking, dietary patterns, alcohol consumption, and self-reported hyperlipidemia, which could have a significant effect on non-HDL-C and lead to errors. However, data such as gender and age, which may also generate errors, were included, and we subsequently performed additional sensitivity analyses by adjusting the model for confounders and for patients with hypertension, heart disease, diabetes mellitus, and other diseases that are closely related to blood lipids.

The European and U.S. Dialysis Patient Guidelines Committee recommends maintaining non-HDL-C levels below 130 mg/dL (3.4 mmol/L), particularly among individuals with elevated fasting triglyceride concentrations. Previous research also suggests that keeping adult non-HDL cholesterol levels below 130 mg/dL (3.4 mmol/L) is best for lowering the risk of cardiovascular death (37–39). Specifically, for every 0.8 mmol/L increase in non-HDL-C, there is a 19% higher risk of cardiovascular death in men and an 11% higher risk in women (40). Additionally, individuals with normal levels of LDL-C face a 32% increased risk of cardiovascular events when their non-HDL-C levels exceed 130 mg/dL (41).

### 2.4 Statistical analysis

This study conducted an initial analysis of non-HDL-C through descriptive statistics, including the calculation of its mean and variance, and examined variations between groups of differing debilitation grades using the univariate analysis of variance (ANOVA) test. Continuous variables were analyzed using ANOVA followed by Fisher’s Least Significant Difference (LSD) *post hoc* test. Categorical variables were analyzed using the chi-square test with standardized residuals, with a threshold of *p* < 0.05 to establish statistical significance. Subsequently, the impact of non-HDL-C on frailty (dichotomous, utilized for all subsequent analyses) was evaluated through univariate logistic regression. To address potential confounding factors, we employed a mixed-factor adjustment approach that integrated variable inflation factor (VIF), Least Absolute Shrinkage and Selection Operator (LASSO) regression, and LightGBM techniques to identify essential relative variables that exhibit both statistical significance and mutual independence within the model. During the modeling process, 70% of the dataset was allocated for model training with 5-fold cross-validation, while the remaining 30% was reserved for final model testing to evaluate the robustness and consistency of the results (24, 25, 35). Furthermore, in order to investigate the nonlinear association between non-HDL-C levels and severity of frailty, we utilized partial dependency plots (PDP) and polynomial regression methodologies within the framework of machine learning. Subsequent subgroup analyses were conducted to assess variations in the impact of non-HDL-C across different subgroups through the creation of interaction terms. To evaluate the heterogeneity between different databases and mitigate the influence of cardiac and metabolic conditions, we employed mixed linear modeling (MixedLM) in conjunction with the FI index to identify the independent relationship between non-HDL-C levels and frailty severity, elucidating a significant role in regulating this biomarker in individuals with frailty. All analyses were performed using Python 3.8.

## 3 Results

### 3.1 Characteristics of study population

Significant variations in age, gender, education, marital status, and race were observed among individuals categorized into non-frail, pre-frail, and frail groups (Table 1). The average age of patients in the frail group was notably higher compared to those in the pre-frail group and the non-frail group (*p* < 0.001). The weakening of physiological reserves brought about by the natural aging process (due to the increased risk of adverse outcomes as a result of aging) impinges on the individual’s state of frailty and seems to explain this phenomenon. In addition, the sex ratio was appropriate in this study to avoid the errors that could be generated. Racial disparities among the three groups were also statistically significant based on chi-square analysis, indicating a need for further investigation (Table 1). Meanwhile, the variables including non-HDL-C, BUN, and serum glucose were found to have statistically significant associations with frailty. Furthermore, the mean BMI of frail participants was notably higher compared to the other two participant groups (*p* < 0.001). Additionally, levels of blood glucose, waist circumference, serum creatinine, and uric acid were significantly elevated in the frail group in comparison to both the pre-frail and non-frail groups (*p* < 0.001). Furthermore, leukocyte counts indicative of inflammatory conditions exhibited notable disparities among the three cohorts, with significantly elevated levels observed in the frail group compared to both the pre-frail and non-frail groups (Table 1).

**Table 1:** Variables and baseline characteristics of the participants.

Variables	Categories	Total	Non-frail	Pre-frail	Frail	*p*-value	*p*-value*	*p*-value**	*p*-value***
Age		72.413 ± 5.378	71.834 ± 5.319	72.237 ± 5.337	72.910 ± 5.424	<0.001	0.015	<0.001	<0.001
Total cholesterol (mg/dL)		188.588 ± 38.115	195.586 ± 36.567	189.448 ± 37.577	184.429 ± 39.017	<0.001	<0.001	<0.001	<0.001
HDL-C (mg/dL)		53.696 ± 14.993	57.731 ± 16.534	53.943 ± 14.615	51.661 ± 14.495	<0.001	<0.001	<0.001	<0.001
Non-HDL-C (mg/dL)		134.892 ± 36.726	137.855 ± 35.307	135.506 ± 36.277	132.768 ± 37.832	<0.001	0.034	<0.001	0.001
Waist circumference (cm)		94.596 ± 15.637	86.939 ± 11.587	93.348 ± 14.936	99.561 ± 16.470	<0.001	<0.001	<0.001	<0.001
BMI (kg/m^2^)		26.178 ± 5.757	23.364 ± 3.838	25.691 ± 5.228	28.047 ± 6.516	<0.001	<0.001	<0.001	<0.001
WBC (10^9^/L)		6.636 ± 1.886	6.339 ± 1.738	6.527 ± 1.828	6.919 ± 1.989	<0.001	0.001	<0.001	<0.001
Hemoglobin (g/dL)		13.771 ± 1.580	13.977 ± 1.508	13.853 ± 1.578	13.567 ± 1.592	<0.001	0.010	<0.001	<0.001
MCV (fL)		91.419 ± 6.373	91.733 ± 6.235	91.583 ± 6.322	91.049 ± 6.487	<0.001	0.441	0.001	<0.001
HCT (%)		40.885 ± 4.568	41.344 ± 4.405	41.055 ± 4.510	40.447 ± 4.685	<0.001	0.037	<0.001	<0.001
PLT (10^9^/L)		211.841 ± 61.601	211.354 ± 58.610	211.191 ± 61.044	212.991 ± 63.592	0.426	0.394	<0.001	0.029
BUN (mg/dL)		17.131 ± 5.938	16.314 ± 4.841	16.814 ± 5.571	17.935 ± 6.728	<0.001	0.001	<0.001	<0.001
Glucose, serum (mg/dL)		109.416 ± 32.973	102.879 ± 24.306	107.468 ± 29.843	114.969 ± 39.069	<0.001	<0.001	<0.001	<0.001
Creatinine, serum (mg/dL)		0.937 ± 0.310	0.894 ± 0.244	0.926 ± 0.284	0.971 ± 0.364	<0.001	<0.001	<0.001	<0.001
Glycosylated hemoglobin (%)		5.876 ± 0.912	5.616 ± 0.624	5.829 ± 0.860	6.053 ± 1.043	<0.001	<0.001	<0.001	<0.001
Uric acid (mg/dL)		5.377 ± 1.448	5.159 ± 1.305	5.351 ± 1.413	5.508 ± 1.539	<0.001	<0.001	<0.001	<0.001
							SR*	SR**	SR***
Gender	Male	4,589 (50.169%)	743 (56.245%)	2,401 (51.712%)	1,445 (45.397%)	<0.001	3.118	1.484	−3.801
	Female	4,558 (49.830%)	578 (43.755%)	2,242 (48.288%)	1738 (54.603%)		−3.128	−1.489	3.814
Race	Asian	4,175 (45.643%)	632 (47.843%)	2,211 (47.620%)	1,332 (41.847%)	<0.001	1.183	1.994	−3.170
	African-American	921 (10.069%)	120 (9.084%)	461 (9.929%)	340 (10.682%)		−1.128	−0.301	1.090
	White people	2,871 (31.387%)	433 (32.778%)	1,424 (30.670%)	1,014 (31.857%)		0.902	−0.873	0.473
	Latino/Hispanic	1,033 (11.293%)	116 (8.781%)	483 (10.403%)	434 (13.635%)		−2.717	−1.806	3.931
	Other race	147 (1.607%)	20 (1.514%)	64 (1.378%)	63 (1.979%)		−0.267	−1.229	1.656
Education	Primary education and below	4,753 (51.962%)	585 (44.285%)	2,356 (50.743%)	1812 (56.927%)	<0.001	−3.871	−1.153	3.886
	Secondary education	1786 (28.512%)	247 (18.698%)	916 (19.729%)	623 (19.573%)		−0.681	0.313	0.060
	Higher education	2,608 (19.526%)	489 (37.017%)	1,371 (29.528%)	748 (23.500%)		5.789	1.297	−5.296
Marital status	Unmarried	5,454 (63.737%)	903 (68.357%)	2,991 (64.419%)	1843 (57.901%)	<0.001	2.587	1.462	−3.433
	Married	2,133 (24.927%)	45 (3.407%)	117 (2.520%)	96 (3.016%)		1.268	−1.220	0.656
	Widowed	739 (8.636%)	109 (8.251%)	390 (8.400%)	314 (9.965%)		−0.776	−1.116	1.848
	Divorced	228 (2.664%)	264 (19.985%)	1,145 (24.661%)	930 (29.218%)		−4.015	−1.227	4.068

Percentages were used for gender, race, education, and marital status. Categorical variable P-value was calculated using chi-square test. Mean ± SD: age, BMI (kg/m^2^), Non-HDL-C (mg/dL), HDL-C (mg/dL), Total cholestero l (mg/dL), WBC (10^9^/L), Hemoglobin (g/dL), MCV (fL), HCT (%), PLT (10^9^/L), BUN (mg/dL), Glucose, serum (mg/dL), Creatinine, serum (mg/dL), Glycosylated hemoglobin (%), and Uric acid (mg/dL). Continuous variable *p*-values were calculated using ANOVA. Categorical variables were tested using chi-square tests and post hoc tests using standardized residuals. *p*-value*: *p*-value based on Fisher’s Least Significant Difference (LSD) post hoc test for Non-frail and Pre-frail. *p*-value**: *p*-value based on Fisher’s LSD post hoc test *p*-values for Non-frail and Frail. *p*-value***: *p*-value for Pre-frail and Frail based on Fisher’s LSD *post-hoc* test. BMI, body mass index; WBC, white blood cell count; MCV, mean erythrocyte volume; HCT, erythrocyte pressure volume; PLT, platelet count; BUN, urea nitrogen. SR*standardized residual of category vs. non-frail. SR**standardized residual of category vs. pre-frail. SR***standardized residual of category vs. frailty, with an absolute value of the standardized residual greater than 2.5 indicating that there is a significant deviation between the observed and expected frequencies in the combination.

### 3.2 Univariate logistic regression analysis

**Figure 2:**
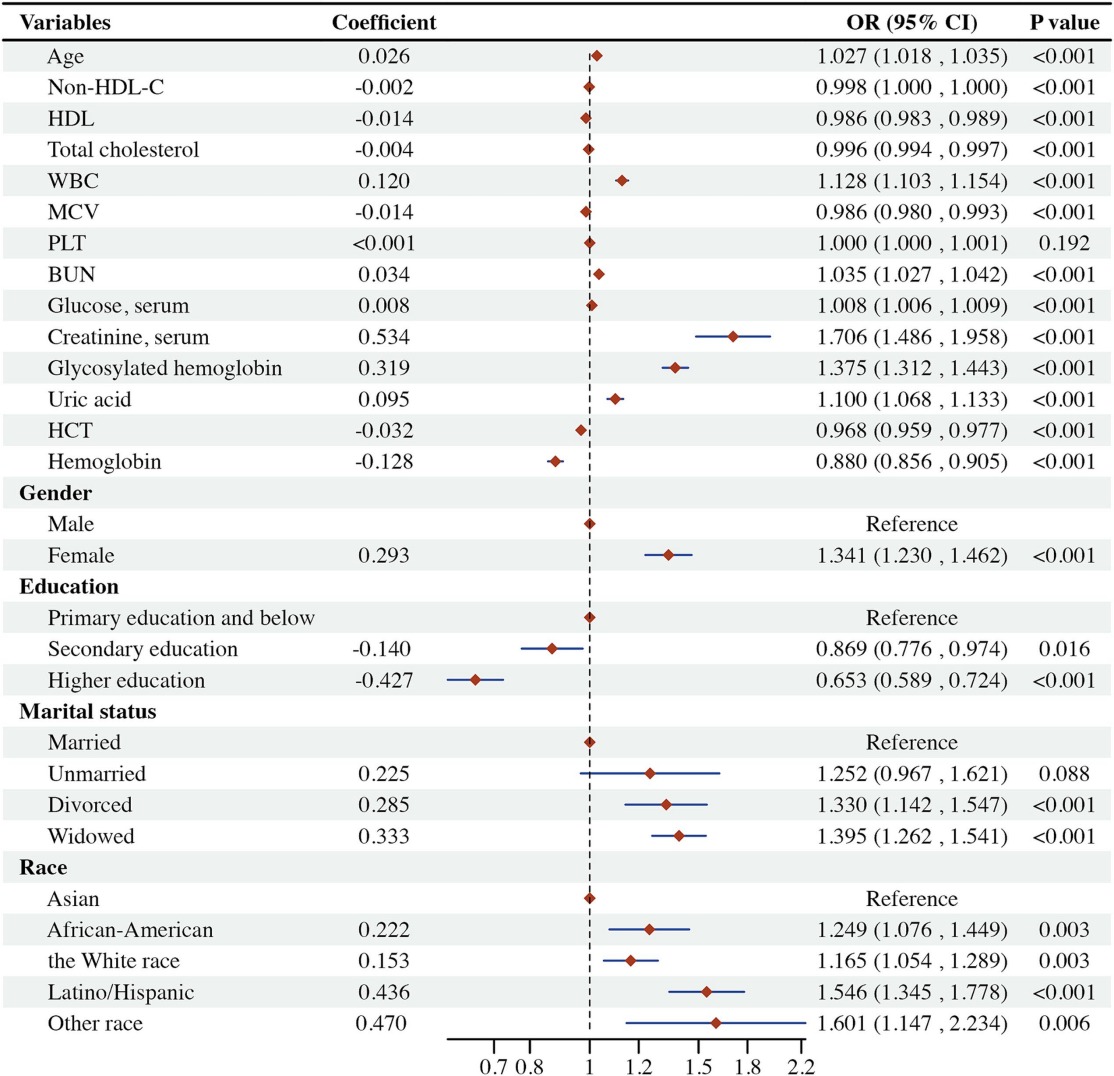
The univariate logistic regression results. BMI, body mass index; WBC, white blood cell count; MCV, mean erythrocyte volume; HCT, erythrocyte pressure volume; PLT, platelet count; BUN, urea nitrogen. 1*, this group was used as a reference for intergroup control.

To investigate the association between non-HDL-C levels and frailty, the pre-frail group was amalgamated with the non-frail group, thereby transforming the frail classification into a dichotomous variable. Univariate logistic regression was employed to examine the relationship between non-HDL-C levels, additional laboratory parameters, gender, age, race, education, BMI, and the onset of frailty. The findings indicated that marital status and level of education up to secondary school were not significant factors in the onset of frailty. Conversely, non-HDL-C levels, mean red blood cell volume, erythrocyte pressure volume, hemoglobin levels, total cholesterol levels, and attainment of higher education were inversely related to the development of frailty. On the other hand, advancing age, waist circumference, white blood cell levels, platelets, urea nitrogen, blood glucose levels, creatinine levels, glycated hemoglobin, marital status as widowed or divorced, race, and BMI levels were positively correlated with the onset of frailty (Figure 2).

### 3.3 Multivariate analysis

**Figure 3:**
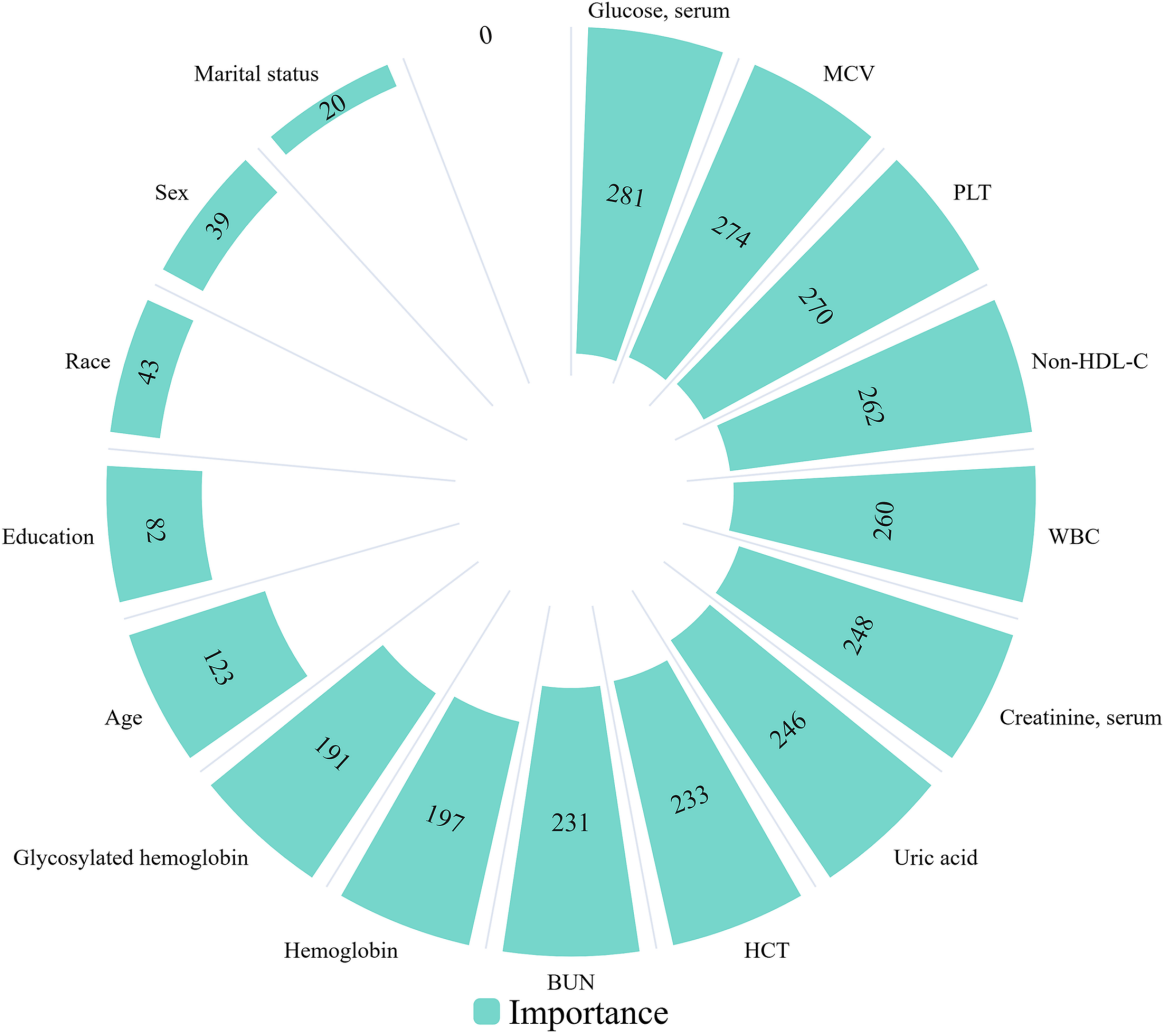
Relative importance of selected variables and corresponding variable importance scores.

As demonstrated in Supplementary Table 1, an analysis of the covariance in the regression model led to the exclusion of two variables with VIF greater than 10, namely HDL-C and total cholesterol. LightGBM exhibited superior capability in capturing intricate relationships between categorical and continuous variables while adjusting for additional confounding factors when compared to logistic regression (31, 42). Consequently, LightGBM was employed to model the relationship between frailty and non-HDL-C, determining the significance of each variable and establishing their order of importance. Subsequently, categorical variables with low significance were excluded, and the coefficients of each characteristic were obtained through binary logistic regression with LASSO regularization. Variables with coefficients of 0 were then removed, resulting in a final set of significant variables including BMI, non-HDL-C, waist circumference, blood glucose, blood creatinine, white blood cells, glycosylated hemoglobin, and hemoglobin for further multivariate analysis (Figure 3).

**Figure 4:**
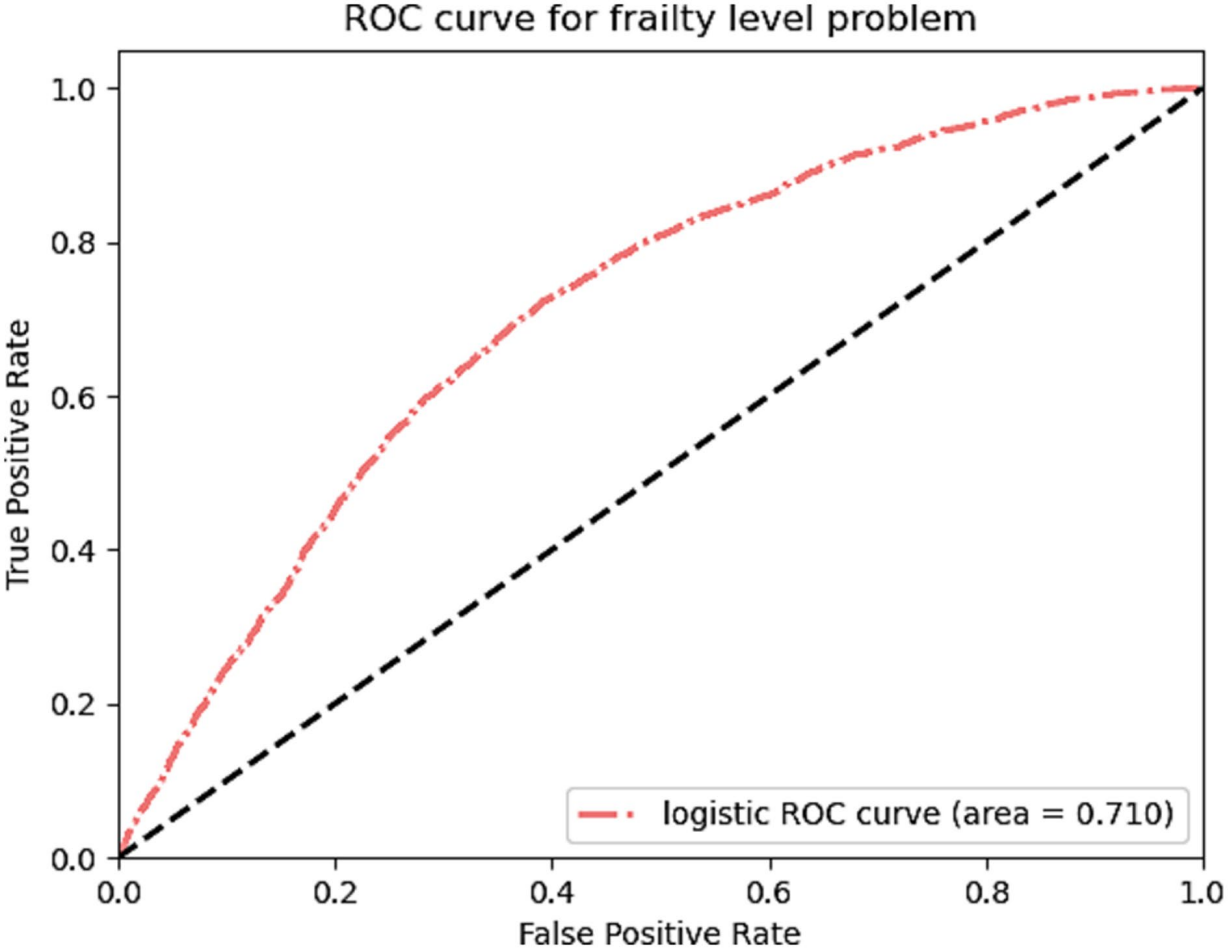
Micro-averaged receiver operating characteristic curves plotted according to multivariate logistic regression.

Following the selection of significant variables, a multinomial logistic regression model was constructed, and Receiver Operating Characteristic (ROC) curves were produced for validation (Figure 4). The result revealed an area under the curve (AUC) value of 0.710 for the ROC curve of the multinomial logistic regression model. This information holds importance in guiding clinical decision-making and improving individualized treatment strategies.

### 3.4 Nonlinear

**Figure 5:**
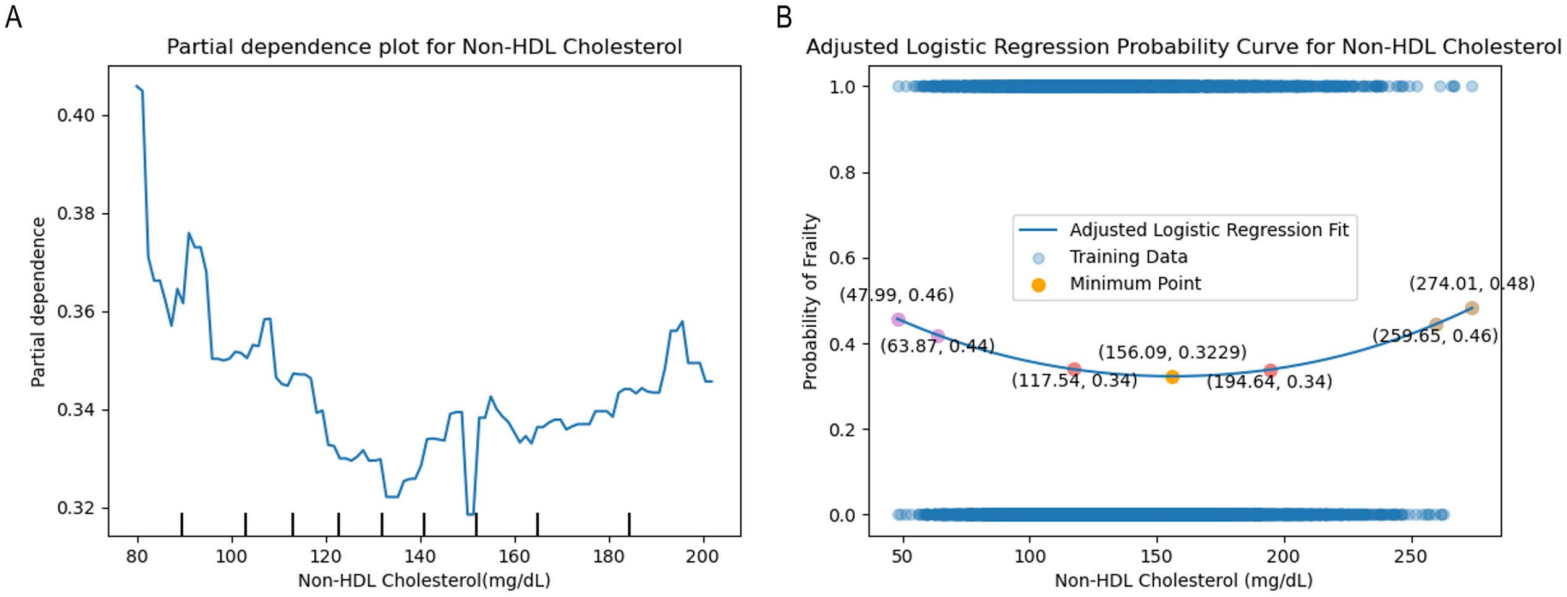
**(A)** The partial dependency plot (PDP) generated by the LightGBM algorithm. **(B)** Second-order polynomial fit curves show a nonlinear trend and curvilinear relationship between non-HDL cholesterol and frailty. Quartiles were used to qualify the range of values, with 25% near the threshold being the ideal range (lower risk of frailty) and 12.5% on either side being the range that would need to be assessed for frailty.

Given the intricate nature of frailty’s etiology and pathophysiological mechanisms, we also investigated the presence of a nonlinear relationship between non-HDL-C levels and frailty. After adjusting for confounding factors, the PDP generated by the LightGBM algorithm was utilized to examine the role of non-HDL-C in predicting frailty. Findings indicated a nonlinear association between non-HDL-C levels and frailty, which remained unaffected by other variables (Figure 5A). The fluctuating curves observed suggest that the predictive value of the model varies with changes in non-HDL-C levels. We further analyzed this nonlinear relationship using a polynomial fitting method and clarified that a second-order polynomial was the most appropriate choice by the Akaike Information Criterion (AIC). Examination of second-order polynomial fit plots suggested a potential U-shaped correlation between non-HDL-C and frailty. The risk of frailty decreased notably until the non-HDL-C levels reached the nadir (156.09 mg/dL), after which a positive correlation was observed (Figure 5B). This U-shaped non-linear relationship highlights the intricate interaction between the variables, underscoring the non-linear nature of the impact of non-HDL-C levels on the frail state. Understanding this complexity is crucial for informing the development of effective clinical prevention and treatment strategies. In addition, we developed reference-worthy ranges of non-HDL-C for controlling the risk of frailty based on quartiles. The risk of frailty was low at 117.54–194.64 mg/dL and high at 47.99–63.87 and 274.01–259.65 mg/dL. Individuals with non-HDL-C levels in the latter range should be promptly assessed for frailty in order to facilitate reversal of the frailty trajectory.

### 3.5 Subgroup, heterogeneity and sensitivity

**Figure 6:**
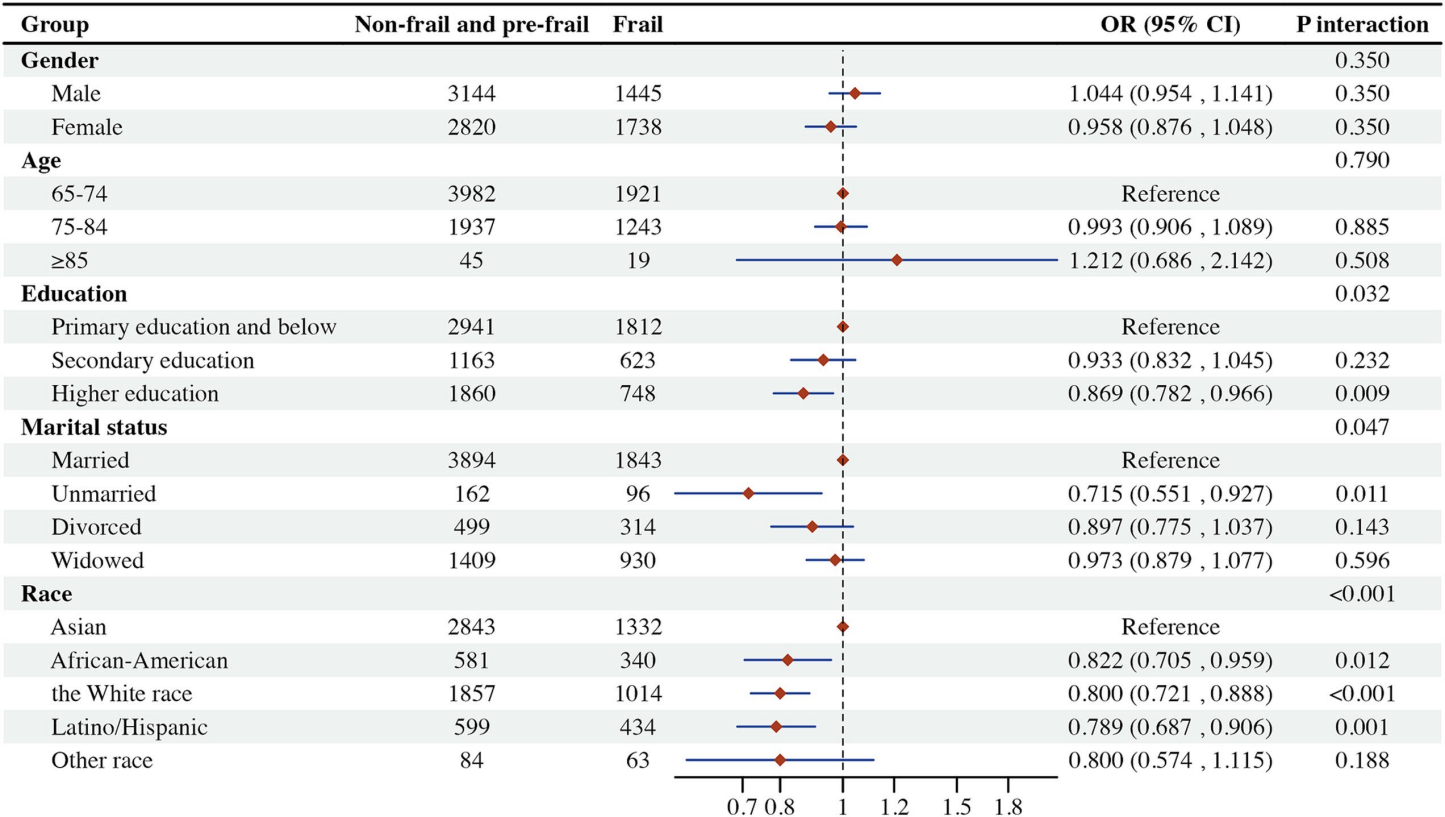
Subgroup analysis and interaction test. 1*, this group was used as a reference for intergroup control.

To better interpret the results, subgroup analyses and interaction tests were performed (43). This information is valuable for informing clinical decision-making and enhancing personalized treatment approaches. The study employed stratification by gender, race, education, and age to assess the generalizability of the association between non-HDL-C levels and frailty across various subgroups within the population. The findings indicated a lack of interaction among subgroups, except non-HDL-C levels interacting with the risk of frailty for race (Figure 6). This result supports the generalizability of population-based conclusions across various subgroups, underscoring their consistency and dependability.

In this study, the MixedLM was employed for the analysis of heterogeneity, a statistical method that incorporates random effects to account for variability in the data. The analysis of the model revealed a Group Variance value of 0.000161 with a standard error of 0.001. This finding suggests that despite originating from disparate international databases, namely those of China and the United States, the data pertaining to debilitation levels analyzed display a reduced level of heterogeneity. This observation indicates a potential high level of uniformity in the processing and measurement of debilitation ratings across different countries, or a similarity in the underlying factors influencing these ratings within these populations.

Moreover, we employed a cross-validation method to ensure consistent sensitivity analysis throughout the modeling process. Specifically, the dataset was partitioned into five subsets, with one serving as the test set and the remaining subsets as the training set. This iterative process of training and testing models was repeated to evaluate the reliability and generalizability of the findings.

Additionally, a further sensitivity analysis was conducted to control for potential confounding factors such as heart disease and metabolic disorders, to assess the independent relationship between non-HDL-C levels and frailty. The findings were in line with previous research, demonstrating a consistent nonlinear association between non-HDL-C levels and frailty.

## 4 Discussion

The significant adverse consequences of frailty pose a substantial medical burden on both patients and their families (2, 44). Various therapeutic strategies have been suggested for addressing frailty. Nevertheless, due to incomplete comprehension of its pathophysiological mechanisms, only modifications in nutritional interventions and exercise rehabilitation have demonstrated some efficacy (2, 45). Despite some focus and investigation on the modulation of lipid metabolism in the management of frailty, the significance of non-HDL-C and its association with frailty has not been adequately acknowledged.

It is widely accepted that elevated non-HDL-C levels may contribute to the onset and progression of frailty. Several potential mechanisms could account for this theory. One such mechanism is that non-HDL-C may impact normal physiological processes through various regulatory pathways that influence cardiovascular well-being (27, 46). Furthermore, an increase in non-HDL-C levels is likely to result in stroke, which can impair cognitive function, reduce self-care abilities, and ultimately diminish quality of life while also increasing economic burden and disease risk (47, 48). In addition, non-HDL-C, encompassing LDL, lipoprotein (a), triglyceride-rich lipoproteins (TRLs), and TRL residues, can infiltrate the arterial wall through the cytosol, leading to damage to the vascular endothelium. This process stimulates the release of proinflammatory factors by nearby cells and interacts with extracellular matrix components, such as proteoglycans, within the endothelium, initiating metabolic and immunoinflammatory responses. This cascade of events attracts monocytes to the site of lesion initiation, promoting their differentiation into macrophages and ultimately resulting in the formation of foam cells. These events contribute to the development of a localized, chronic inflammatory infiltrate over the long term (28, 49, 50), which will disrupt normal bodily functions, potentially leading to decreased muscle mass and function, ultimately resulting in sarcopenia and frailty. Moreover, the association between oxidative stress and increased non-HDL-C is particularly significant. Research indicates that under conditions of heightened oxidative stress, such as those observed in metabolic syndrome, HDL can undergo oxidative modification (22, 51). This modification results in elevated non-HDL levels, which subsequently contribute to the destabilization of vascular endothelial cells. Additionally, oxidative stress impairs the antioxidant function of HDL, primarily mediated by the presence of SOD-1 (22). A clinical study involving 66 elderly individuals without acute or severe chronic illnesses demonstrated a notable reduction in HDL-C levels among participants with frailty and metabolic syndrome (51). Furthermore, within the frailty cohort, SOD-1 exhibited a negative correlation with both total cholesterol and HDL levels. In addition, it is important to consider the potential indirect impact of non-HDL-C on frailty development through its influence on patients’ circadian rhythms as well. A metabolomics investigation revealed that the plasma metabolites exhibiting circadian oscillations were 80% lipid-based (52). Subsequent research delved deeper into lipid metabolites within the human bloodstream, indicating that heightened levels of total cholesterol and total lipids could potentially disrupt circadian rhythms, resulting in increased glucose, insulin, and triglyceride concentrations, hastened lipid storage, and ultimately contributing to the onset of obesity, muscle atrophy, reduced physical activity levels, and heightened susceptibility to frailty (53). Hence, it is imperative to underscore the predictive significance of non-HDL cholesterol and the necessity of controlling its concentrations in the assessment and treatment of frailty to facilitate timely identification and mitigation of frailty.

Nevertheless, our study identified a non-linear U-shaped association between non-HDL-C levels and the risk of frailty. Before reaching the nadir (156.09 mg/dL), higher non-HDL-C levels were inversely related to frailty risk. Within this range, elevated non-HDL-C levels were found to be beneficial for older adults. However, beyond this point, a positive correlation was observed between non-HDL-C levels and frailty, with non-HDL-C becoming a detrimental factor in the progression of frailty in patients. Indeed, previous research on the correlation between cholesterol levels and frailty has also found that dyslipidemia may have the potential to mitigate or even reverse the decline associated with aging. Abnormal lipid levels in middle-aged individuals may transition from being a risk factor to a protective factor in later life (54). Moreover, further studies have indicated that total cholesterol and LDL levels, as markers of metabolic changes and reversal of metabolism, are inversely related to mortality (55, 56). The underlying mechanisms responsible for the paradoxical relationships in older adults have not been fully elucidated to date. Some theories propose that traditional lipid risk factors may exhibit a protective influence, potentially stemming from the inherent physiology of the aging process and the occurrence of metabolic changes or transitions during specific stages of advanced age (55). This concept of reverse metabolism offers insight into the etiology of malnutrition and inflammation in elderly individuals and guides for exploring the underlying biological mechanisms contributing to the onset of frailty.

Subgroup and interaction analyses confirmed of the robustness and reliability of our findings. Specifically, the subgroup analyses revealed that individuals of African-American, White, and Latino/Hispanic descent exhibited lower likelihoods of frailty compared to individuals of Asian descent. This disparity may be attributed to potential biases stemming from the relatively low personal income levels among Chinese individuals in 2011 and 2015, as well as the predominantly rural composition of the data within the CHARLS database. Consequently, further in-depth clinical investigations are warranted to elucidate these findings.

In contrast to prior research, the current study demonstrates unique strengths and innovations. Firstly, the study utilized real population data from the United States and China, encompassing a substantial sample size of 8,554 individuals aged 65 years or older, thus constituting a large-scale cross-sectional study. Secondly, the study employed the LightGBM algorithm and LASSO regression, recognized as highly effective statistical techniques for variable screening and validation. Furthermore, in order to more accurately represent the intricate association between non-HDL-C and frailty, a second-order polynomial was employed for curve fitting, revealing a U-shaped non-linear relationship between attenuation and non-HDL-C levels. When non-HDL-C levels are below 156.09 mg/dL, there is a protective association between non-HDL-C and frailty, transitioning to a detrimental relationship above this level. Notably, our findings also suggest that the risk of frailty is low at 117.54–194.64 mg/dL and high at 47.99–63.87 as well as 274.01–259.65 mg/dL, and that such populations should be assessed for frailty in a timely manner to facilitate the development of individualized intervention programs to ultimately reverse the trajectory of frailty. Subsequently, subgroup analyses and interaction tests were conducted to confirm the validity of the findings and broaden their generalizability.

## 5 Limitations

Despite the promising and dependable results, our study still has several limitations. Firstly, the cross-sectional design of this study made it difficult to determine a possible causal relationship between non-HDL-C and frailty. We suggest that more adequate prospective cohort studies should be conducted in the future to improve the reliability of our conclusions. Furthermore, it is recommended that future research investigate longitudinal relationships between fluctuations in non-HDL-C levels and individual frailty status to enhance the development of personalized intervention approaches. Secondly, the samples used in this study were drawn from population survey data in the United States and China, raising questions about the generalizability of our findings to other countries and regions. In addition, the reliance on self-reported data in this study may introduce subjective bias and recall bias. Lastly, it is worth noting that older adults frequently experience chronic diseases such as chronic liver disease and chronic kidney disease. Our study incorporated hypertension, diabetes, stroke, and heart disease in the evaluation of frailty. However, there remain certain disease biomarkers that have not been accounted for and adjusted. In addition, it is important to acknowledge that the frailty assessment in this study relies on a proprietary frailty scale, potentially introducing bias.

## 6 Conclusion

Overall, the findings of this cross-sectional study utilizing data from the NHANES and CHARLS databases indicate a nuanced, nonlinear association between non-HDL-C and frailty. Elevated non-HDL cholesterol levels, which are considered harmful in middle-aged adults, may serve as protective factors against frailty in older adults when the levels are in the range of 117.54–194.64 mg/dL. Whereas, when non-HDL-C levels are in the range of 47.99–63.87 as well as 274.01–259.65 mg/dL, it is timely to carry out a frailty assessment in order to enable individualized clinical interventions to ameliorate or reverse the state of frailty. The elucidation of this intricate nonlinear association holds significant implications for clinical practice and the care of frail patients.

## Data Availability

Publicly available datasets were analyzed in this study. This data can be found here: the datasets used and analyzed during the current study are available in the NHANES (https://www.cdc.gov/nchs/nhanes/index.htm) and CHARLS (https://charls.pku.edu.cn/). All analyzed data during the current study are available from the corresponding author on reasonable request.
