# Correction: U-shaped relationship between frailty and non-HDL-cholesterol in the elderly: a cross-sectional study

**DOI:** 10.3389/fnut.2025.1650863

**Published:** 2025-07-23

**Authors:** Yu Pan, Yan Yuan, Juan Yang, Zhu Qing Feng, Xue Yin Tang, Yi Jiang, Gui Ming Hu, Jiang Chuan Dong

**Affiliations:** ^1^Department of Geriatrics, The Second Affiliated Hospital of Chongqing Medical University, Chongqing, China; ^2^Department of Integrated of Chinese and Western Medicine, The First Affiliated Hospital of Chongqing Medical University, Chongqing, China; ^3^Oncology Treatment Center of Traditional Chinese Medicine, Affiliated Cancer Hospital of Chongqing University, Chongqing, China

**Keywords:** frailty, non-HDL-cholesterol, cross-sectional study, CHARLS, NHANES

A correction has been made to **Methods**, *Study design*, Paragraph 2. This sentence previously stated:

“This study initially enrolled 82,611 participants from multiple survey cycles, including the NHANES surveys from 2009 to 2010, 2011 to 2012, 2013 to 2014, 2015 to 2016, 2017 to 2018, and the CHARLS 2011 and 2015 surveys. According to the standards of the World Health Organization and the United Nations, people over 65 years old are defined as the elderly. Participants with missing or potentially anomalous relevant data or below the age of 65 were excluded. Additionally, it has been shown in prior research that despite potential missing data among participants, frailty can still be identified through a minimum of 75% FI assessments, even if the specific correlates of frailty may vary. As a result, individuals with a significant amount of missing data and who did not complete at least 75% FI assessments were excluded from the study (7, 8). Ultimately, a total of 8,554 participants were included in the study (Figure 1).”

The corrected sentence appears below:

“This study initially enrolled 88,365 participants from multiple survey cycles, including the NHANES surveys from 2009 to 2010, 2011 to 2012, 2013 to 2014, 2015 to 2016, 2017 to 2018, and the CHARLS 2011 and 2015 surveys. According to the standards of the World Health Organization and the United Nations, people over 65 years old are defined as the elderly. Participants with missing or potentially anomalous relevant data or below the age of 65 were excluded. Additionally, it has been shown in prior research that despite potential missing data among participants, frailty can still be identified through a minimum of 75% FI assessments, even if the specific correlates of frailty may vary. As a result, individuals with a significant amount of missing data and who did not complete at least 75% FI assessments were excluded from the study (7, 8). Ultimately, a total of 9,147 participants were included in the study (Figure 1).”

The original article has been updated.

